# Complete genome sequence of bacteriophage vB_YenP_AP5 which infects *Yersinia enterocolitica* of serotype O:3

**DOI:** 10.1186/1743-422X-11-188

**Published:** 2014-10-28

**Authors:** Carlos G Leon-Velarde, Andrew M Kropinski, Shu Chen, Arash Abbasifar, Mansel W Griffiths, Joseph A Odumeru

**Affiliations:** Laboratory Services Division, University of Guelph, Guelph, ON N1H 8J7 Canada; Department of Pathobiology, University of Guelph, Guelph, ON N1G 2W1 Canada; Department of Molecular and Cellular Biology, University of Guelph, Guelph, ON N1G 2W1 Canada; Canadian Research Institute for Food Safety, University of Guelph, Guelph, ON N1G 2W1 Canada; Department of Food Science, University of Guelph, Guelph, ON N1G 2W1 Canada

## Abstract

**Background:**

Bacteriophage vB_YenP_AP5 is a lytic bacteriophage capable of infecting *Yersinia enterocolitica* strains of serotype O:3, an epidemiologically significant serotype within this bacterial species that causes yersiniosis in humans. This work describes the complete genome sequence of this phage.

**Results:**

The genome consists of linear double-stranded DNA of 38,646 bp, with direct terminal repeats of 235 bp in length, and a GC content of 50.7%. There are 45 open reading frames which occupy 89.9% of the genome. Most of the proteins encoded by this virus exhibit sequence similarity to *Yersinia* phage φYeO3-12 and *Salmonella* phage φSG-JL2 proteins.

**Conclusions:**

Genomic and morphological analyses place the bacteriophage vB_YenP_AP5 in the *T7likevirus* genus of the subfamily *Autographivirinae* within the family *Podoviridae*.

## Background

*Yersinia enterocolitica,* a facultatively anaerobic, Gram-negative, non-sporulating, short bacillus, is an important zoonotic pathogen leading to human and animal enteric infection [1]. Among the species of the genus *Yersinia*, *Y. enterocolitica* is considered highly heterogeneous and is grouped into a biochemical scheme composed of six biotypes divided into three lineages: avirulent strains belonging to biotype 1A, highly pathogenic strains of biotype 1B, and weakly pathogenic strains of biotypes 2–5 that do not kill mice [2, 3]. Most strains associated with yersiniosis belong to bioserotypes 1B/O:8, 2/O:5,27, 2/O:9, 3/O:3, and 4/O:3, with the latter being the most common in Europe, Japan, Canada, and the USA [1, 4]. Although several yersiniophages have been described for typing *Y. enterocolitica* [5–8], few have been studied in detail via whole genome sequencing. To date, phage φYeO3-12 displaying specificity for *Y. enterocolitica* O:3 [9], phage PY54 exhibiting a host range restricted to *Y. enterocolitica* O:5 and O:5,27 [10], phage φR1-37 with a broader host range within *Y. enterocolitica* [11] *,* and PY-100 exhibiting a broad host range restricted to the genus *Yersinia* [12] *,* have been described. Given the considerable interest in bacteriophages because of their potential use as typing, diagnostic, therapeutic, decontaminating, and bio-control agents, our research is aimed at isolating and characterizing novel yersiniophages in order to expand the repertoire of phages available for targeting clinically significant *Y. enterocolitica* bioserotypes. In this manuscript we report the morphology, genome sequence, and transcriptomic analysis of phage vB_YenP_AP5 (hereafter referred to as AP5).

## Results and discussion

### Isolation and host range

Analysis of preliminary treated sewage resulted in the initial isolation of 12 phages infecting *Y. enterocolitica* strains. From these, AP5 was chosen for detailed study because of its ability to infect *Y. enterocolitica* strains of serotype O:3. The host range of AP5 was determined using 60 strains belonging to ten *Yersinia* species at 25°C and at 37°C. The results (Table 1) show that AP5 can form plaques only on *Y. enterocolitica* serotypes O:3, O:2, and O:1 (serotypes with an O antigen known to contain 6-deoxy-L-altropyranose). Other serotypes of *Y. enterocolitica* as well as other species within the genus *Yersinia* were unaffected by the presence of phage AP5. Additionally none of the *Escherichia coli*, *Salmonella*, or *Listeria* species strains tested were infected by this bacteriophage (data not shown). Analysis of *Y. enterocolitica* O:3 rough mutants YeO3-R1 and YeO3-R2 [13] (which are missing the O antigen), were not sensitive to AP5. YeO3-OC, a *Y. enterocolitica* O:3 deletion mutant (Δ*wzx-wbcQ*) which is missing the entire core operon yet produces O antigen [14], was sensitive to AP5. In contrast, its derivative YeO3-OCR, a rough mutant which is also missing the entire core operon yet is unable to produce O antigen [14] was not sensitive to phage AP5. These results indicate the host receptor for phage AP5 lies within the O antigen of the lipopolysaccharide of *Yersinia enterocolitica* O:3 strains, and suggests the O side chain of this serotype (6-deoxy-L-altropyranose) is involved.

**Table 1:** **Bacterial strains used in this study; and host range of AP5 on 60** ***Yersinia*** **strain**s **at 25°C and at 37°C**

***Yersinia***strains	Description/Source	Biotype ^1^	Serotype	Degree of lysis ^2^
*Y. aldovae* Z1	Unknown, Canada			N
*Y. aldovae* Z2	Unknown, Canada			N
*Y. bercovieri* Z3	Unknown, Canada			N
*Y. bercovieri* Z4	Unknown, Canada			N
*Y. enterocolitica* gk132	Unknown		O:1	2+
*Y. enterocolitica* JDE029	Human, Finland		O:1	3+
*Y. enterocolitica* gk2943	Unknown, Finland		O:2	3+
*Y. enterocolitica* gk1142	Hare, Finland		O:2	N
*Y. enterocolitica* K3	Patient isolate, Canada	3	O:3	2+
*Y. enterocolitica* K9	Patient isolate, Canada	3	O:3	3+
*Y. enterocolitica* K10	Patient isolate, Canada	3	O:3	3+
*Y. enterocolitica* K11	Patient isolate, Canada	3	O:3	3+
*Y. enterocolitica* K12	Patient isolate, Canada	3	O:3	2+
*Y. enterocolitica* K2	Patient isolate, Canada	4	O:3	3+
*Y. enterocolitica* K6	Patient isolate, Canada	4	O:3	3+
*Y. enterocolitica* A	Patient isolate, Canada	4	O:3	3+
*Y. enterocolitica* B	Patient isolate, Canada	4	O:3	1+
*Y. enterocolitica* K1	Patient isolate, Canada	4	O:3	2+
*Y. enterocolitica* 6471/76 (YeO3)	Patient isolate, Finland	4	O:3	3+
*Y. enterocolitica* 6471/76-c (YeO3-c)	plasmid (pYV) cured derivative of YeO3 [15]	4	O:3	2+
*Y. enterocolitica* YeO3-R1	Spontaneous rough derivative of YeO3-c [13]	4	O:3	N
*Y. enterocolitica* YeO3-R2	Spontaneous rough derivative of YeO3 [13]	4	O:3	N
*Y. enterocolitica* YeO3-OC	Δ(*wzx-wbcQ*) derivative of YeO3, mutant missing outer core operon [14]	4	O:3	3+
*Y. enterocolitica* YeO3-OCR	Δ(*wzx-wbcQ*) spontaneous rough derivative of YeO3-OC [14]	4	O:3	N
*Y. enterocolitica* E	Patient isolate, Canada	1A	O:5	N
*Y. enterocolitica* F	Patient isolate, Canada	1A	O:5	N
*Y. enterocolitica* C	Patient isolate, Canada	2	O:5,27	N
*Y. enterocolitica* D	Patient isolate, Canada	2	O:5,27	N
*Y. enterocolitica* K5	Patient isolate, Canada	2	O:5,27	N
*Y. enterocolitica* K7	Patient isolate, Canada	2	O:5,27	N
*Y. enterocolitica* K8	Patient isolate, Canada	2	O:5,27	N
*Y. enterocolitica* ATCC 9610	Patient isolate, USA	1	O:8	N
*Y. enterocolitica* ATCC 23715	Patient isolate, USA	1	O:8	N
*Y. enterocolitica* ATCC 27729	Patient isolate, Belgium	1	O:8	N
*Y. enterocolitica* I	Patient isolate, Canada	2	O:8	N
*Y. enterocolitica* J	Patient isolate, Canada	2	O:8	N
*Y. enterocolitica* K4	Patient isolate, Canada	2	O:8	N
*Y. enterocolitica* K20	Patient isolate, Canada	2	O:8	N
*Y. enterocolitica* K21	Patient isolate, Canada	2	O:8	N
*Y. enterocolitica* G	Patient isolate, Canada	2	O:9	N
*Y. enterocolitica* H	Patient isolate, Canada	2	O:9	N
*Y. enterocolitica* K13	Patient isolate, Canada	2	O:9	N
*Y. enterocolitica* K14	Patient isolate, Canada	2	O:9	N
*Y. enterocolitica* K15	Patient isolate, Canada	2	O:9	N
*Y. frederiksenii* Q	Unknown, Canada			N
*Y. frederiksenii* S	Unknown, Canada			N
*Y. intermedia* M	Unknown, Canada			N
*Y. intermedia* N	Unknown, Canada			N
*Y. kristensenii* Y	Unknown, Canada			N
*Y. kristensenii* X	Unknown, Canada			N
*Y. kristensenii* ATCC 33639	Hare, unknown			N
*Y. mollaretii* T	Unknown, Canada			N
*Y. mollaretii* U	Unknown, Canada			N
*Y. pseudotuberculosis* K	Patient isolate, Canada		I	N
*Y. pseudotuberculosis* L	Patient isolate, Canada		I	N
*Y. rohdei* V	Unknown, Canada			N
*Y. rohdei* W	Unknown, Canada			N
*Y. ruckeri* O	Unknown, Canada			N
*Y. ruckeri* P	Unknown, Canada			N
*Y. ruckeri* ATCC 29473	Rainbow trout, USA			N

^1^Wauters et al. [2].

^2^Degree of lysis: 4+, complete lysis; 3+ clearing throughout but with faint hazy background, 2+ substantial turbidity throughout cleared zone, 1+ a few individual plaques; N: No effect of phage on bacterial growth as described by Kutter [16].

### Morphology

**Figure 1:**
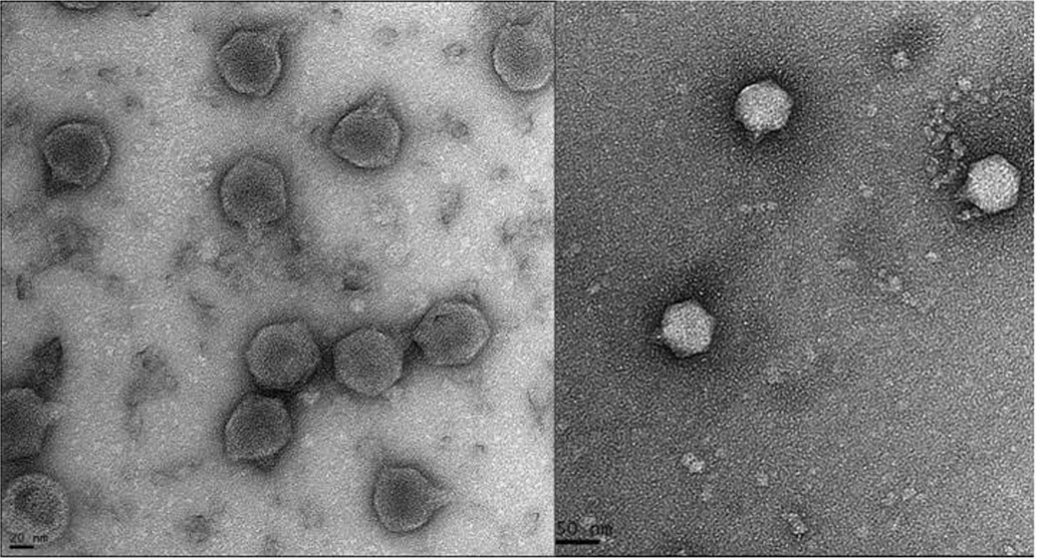
**Electron micrographs of phage AP5.** The phage has been negatively stained with 2% potassium phosphotungstate. AP5 is shown at 50,000× magnification or 150,000× magnification. Scale bar indicates size in nm.

AP5 was negatively stained and examined by transmission electron microscopy (Figure 1). The head is icosahedral in shape exhibiting T7 symmetry of approximately 55.0 nm in diameter. The phage particles are each decorated with a short non-contractile tail of approximately 12.0 nm in length and 8 nm in width. Collectively, these morphological features indicate that this virus belongs to the family *Podoviridae*.

### General features of the AP5 genome

**Figure 2:**
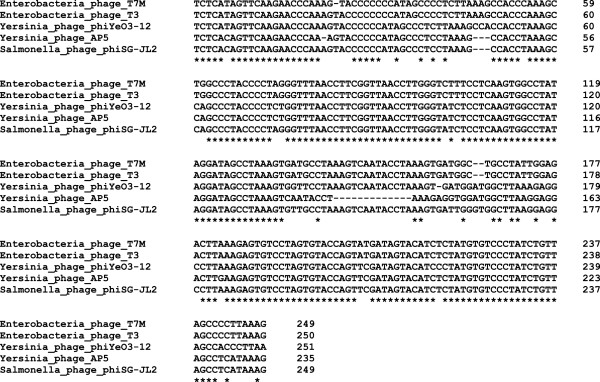
**Multiple sequence alignment of the direct terminal repeats of phage AP5 and selected members of the** ***T7likevirus*** **genus.** Multiple sequence alignment of direct terminal repeat sequences was performed using Clustal Omega [22]. Positions which have a single, fully conserved base pair are indicated by an asterisk (*).

The DNA sequence of the phage AP5 consists of linear double stranded DNA of 38,646 bp in length. The size of this phage correlates well with other T7-like phage members, which range from 37.4 kb (*Pseudomonas* phage gh-1) to 45.9 kb (*Erwinia* phage Era103 [17]. The genomes of T7-like phages typically contain direct terminal repeats (DTRs) that are used during genome replication and packaging [18]. The lengths of the DTRs of AP5 (235 bp) are in agreement with the reported lengths for members of the T7 group, for example phage *Salmonella* phage φSG-JL2 and *Yersinia* phage φYeO3-12 have DTRs of 230 bp and 232 bp, respectively [9], whereas Enterobacteria phage T7 has DTRs of 160 bp [19]. Moreover, an alignment of the DTR sequences of phage AP5 and representative members of the *T7likevirus* genus show a high degree of conservation (Figure 2). Phage AP5 has also an overall genomic guanine plus cytosine (GC) content of 50.7%, compared to 48.5 ± 1.5 mol% for its host [20]. The GC contents of the common representatives of the T7 group, T7 (accession no. V01146.1 [complete sequence of 39,937 bp]) and T3 (accession no. NC_003298.1 [complete sequence of 38,208 bp]), are 48.4% and 49.9%, respectively. The GC content of phage AP5 is in agreement with other T7-like phages which range from 46.2 - 62.3% [21].

### Open reading frames and comparative genomics

**Figure 3:**
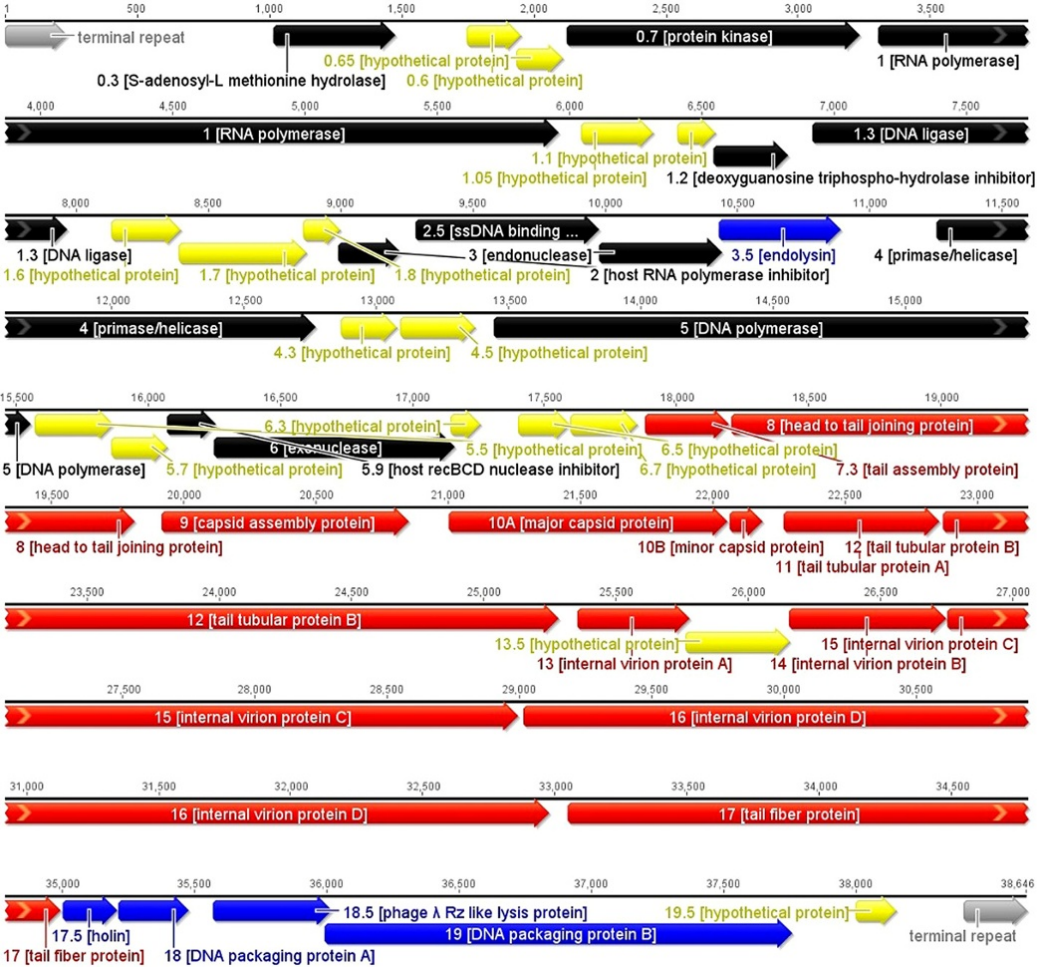
**Genetic and physical map of phage AP5.** Predicted genes are arranged in the direction of transcription. Genes involved in nucleotide metabolism, DNA replication, and recombination are shown in black. Genes involved in phage assembly are depicted in red. Genes involved in DNA packaging and host lysis are shown in blue. Genes encoding hypothetical proteins with unassigned function are shown in yellow. The genetic map was created using *EMBOSS*[27].

**Figure 4:**
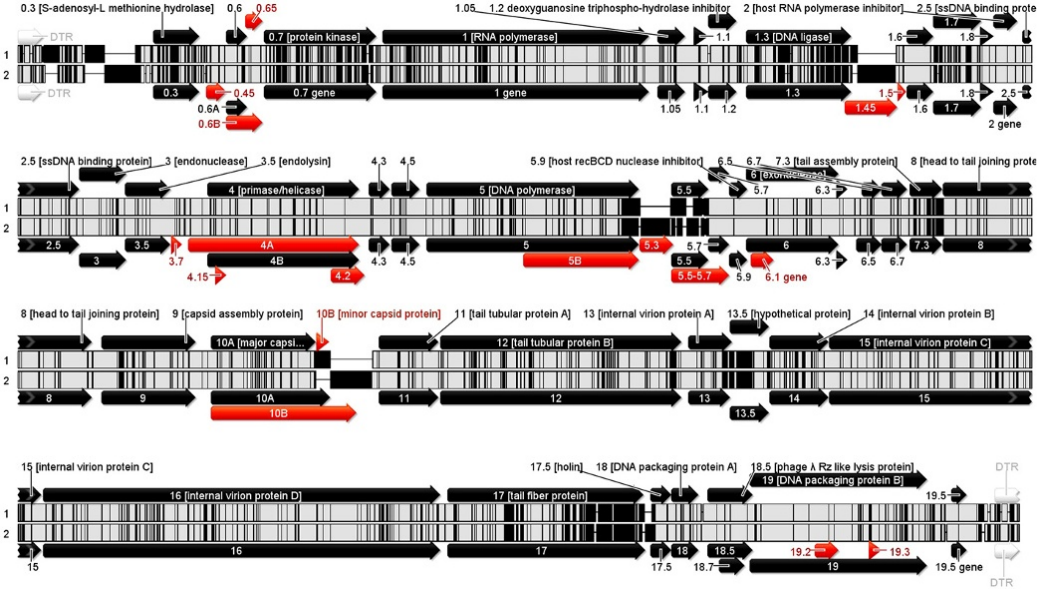
**Progressive mauve alignment of phage AP5 with** ***Yersinia*** **phage φYeO3-12.** Inner tracks show regions of DNA sequence similarity (white) interspersed with regions where no sequence similarity exists (black) [26]. The gene arrangement of AP5 (top) and φYeO3-12 (bottom) is displayed in the direction of transcription. Genes depicted in red indicate genes that are dissimilar and or missing from either genome.

The genome of AP5 was scanned for open reading frames (ORF) using computational software. A total of 34,743 nucleotides were involved in the coding of 45 ORFs with sizes ranging from 113 to 3,962 nucleotides (Table 2). The temporal and functional distributions of genes are tightly organized and packed close to each other so that they occupy 89.9% of the genome (Figure 3). The initiation codon ATG is present in 93.3% of the protein-coding genes. Only two other initiation codons occur, TTG and GTG at a frequency of 0.5%, and 0.2%, respectively. All predicted protein-coding genes were screened using BLASTP and Psi-BLAST algorithms against the non-redundant protein database at NCBI. From the 45 coding sequences (CDSs) of AP5, 30 (66.6%) have assigned function, and 15 (33.3%) are similar to proteins of unknown function. While the great majority of the homologs are to proteins of *Yersinia* phage φYeO3-12 (26), examples of primary sequence similarity to *Salmonella* phage φSG-JL2 (12), *Enterobacteria* phages T3 and T7 (6,1) and *Klebsiella* phage KP32 (1), exist. All of these phages are members of the *T7likevirus* genus. No function can be speculated about the hypothetical proteins of AP5 without further study. Based upon overall protein homology determined using CoreGenes [23, 24], AP5 shares 42 (76.4%), similar proteins with *Enterobacteria* phage T7 and *Enterobacteria* phage T3, 43 (78.2%) similar proteins with *Salmonella* phage φSG-JL2, and 43 (72.9%) similar proteins with *Yersinia* phage φYeO3-12. Collectively these results indicate that AP5 is a member of the *Autographivirinae*, specifically a member of the *T7likevirus* genus [25]. Thus, the T7 gene nomenclature was adopted for naming the genes of AP5. Since at the protein level phage AP5 showed the greatest sequence identity with *Yersinia* phage φYeO3-12 proteins, the genomes of the two phages were compared using progressive Mauve [26] (Figure 4). The gene arrangement of essential genes is collinear, highly conserved, and only some genes coding for hypothetical proteins present in φYeO3-12 are dissimilar or absent in AP5. The pairwise % identity of the phage AP5 genome to *Yersinia* phage φYeO3-12 genome was estimated at 89.6%.

**Table 2:** **Yersiniophage AP5 gene annotations**

Gene name	ORF	Product	Start (bp)	Stop (bp)	pI	Protein mass (Da)	Function or Similarity	Evidence or Organism	Homolog (Accession number)	Id (%)	BlastP E-value
**-**	-	regulatory element	1	235	-	-	terminal repeat	-	-	-	-
**-**	-	regulatory element	550	580	-	-	promoter (sequence similarity to host promoter)	-	-	-	-
***0.3***	*ORF1*	S-adenosyl-L-methionine hydrolase	1,017	1,475	5.94	17171.56	DNA replication, repair and nucleotide metabolism	*Yersinia* phage phiYeO3-12	NP_052065	92.1	1.94e^-101^
***0.6***	*ORF2*	hypothetical protein	1,748	1,951	10.5	7878.43	unknown	*Yersinia* phage phiYeO3-12	NP_052068	98.5	2.31e^-39^
***0.65***	*ORF3*	hypothetical protein	1,938	2,108	11.4	6546.78	unknown	Enterobacteria phage T3	NP_523299	98.0	1.98e^-27^
***0.7***	*ORF4*	protein kinase	2,123	3,232	7.19	42424.34	host transcription shutoff and Col Ib exclusion	*Salmonella* phage phiSG-JL2	YP_001949749	93.2	0
***1***	*ORF5*	RNA polymerase	3,303	5,957	7.10	98841.61	RNA replication, transcription and modification	Enterobacteria phage T3	NP_523301	99.3	0
***1.05***	*ORF6*	hypothetical protein	6,044	6,316	9.22	10529.28	unknown	Enterobacteria phage T3	NP_523302	92.2	2.75e^-53^
***1.1***	*ORF7*	hypothetical protein	6,409	6,549	10.93	5887.54	unknown	*Yersinia* phage phiYeO3-12	NP_052073	95.7	1.26e^-22^
***1.2***	*ORF8*	deoxyguanosine triphospho-hydrolase inhibitor	6,549	6,824	7.01	10433.64	hydrolyzes dGTP and may affect cellular pool of dGTP	*Salmonella* phage phiSG-JL2	YP_001949753	97.8	5.40e^-60^
***1.3***	*ORF9*	DNA ligase	6,919	7,965	5.16	39618.14	DNA replication, recombination, and repair	*Salmonella* phage phiSG-JL2	YP_001949754	92.5	0
***1.6***	*ORF10*	hypothetical protein	8,135	8,392	11.20	9893.68	unknown	*Yersinia* phage phiYeO3-12	NP_052078	100	1.26e^-53^
***1.7***	*ORF11*	hypothetical protein	8,392	8,871	9.38	17869.64	unknown	*Yersinia* phage phiYeO3-12	NP_052079	84.9	7.83e^-95^
***1.8***	*ORF12*	hypothetical protein	8,858	8,995	5.14	5267.92	unknown	*Yersinia* phage phiYeO3-12	NP_052080	100	2.81e^-23^
***2***	*ORF13*	Host RNA polymerase inhibitor	8,992	9,228	4.85	8839.87	inhibition of host RNA Polymerase	*Salmonella* phage phiSG-JL2	YP_001949759	100	2.40e^-27^
***2.5***	*ORF14*	ssDNA binding protein	9,281	9,979	4.80	25965.80	helix-destabilizing protein	*Yersinia* phage phiYeO3-12	NP_052082	98.7	2.40e^-167^
***3***	*ORF15*	endonuclease	9,979	10,440	9.58	17725.54	endonuclease I	Enterobacteria phage T7M	AFQ97046	98.0	1.14e^-103^
***3.5***	*ORF16*	endolysin	10,433	10,888	9.03	16900.28	N-acetylmuramoyl-L-alanine amidase	*Yersinia* phage phiYeO3-12	NP_052084	100	9.21e^-109^
***4***	*ORF17*	primase/helicase	11,255	12,769	5.11	55882.14	DNA replication	*Yersinia* phage phiYeO3-12	NP_052088	99.8	0
***4.3***	*ORF18*	hypothetical protein	12,865	13,077	10.00	7762.16	unknown	Enterobacteria phage T3	NP_523318	98.6	3.66e^-39^
***4.5***	*ORF19*	hypothetical protein	13,090	13,374	9.89	10749.39	unknown	*Yersinia* phage phiYeO3-12	NP_052092	100	1.26^e-62^
***5***	*ORF20*	DNA polymerase	13,442	15,556	6.42	79875.05	DNA replication	*Salmonella* phage phiSG-JL2	YP_001949769	99.0	0
***5.5***	*ORF21*	hypothetical protein	15,573	15,869	5.53	11022.78	unknown	*Klebsiella* phage KP32	YP_003347541	58.3	7.72^e-25^
***5.7***	*ORF22*	hypothetical protein	15,866	16,075	9.81	7260.42	unknown	*Yersinia* phage phiYeO3-12	NP_052098	100	5.77^e-42^
***5.9***	*ORF23*	host recBCD nuclease inhibitor	16,072	16,254	3.1	6742.48	Inhibits host recBCD nuclease	*Yersinia* phage phiYeO3-12	NP_072071	98.3	1.08^e-33^
***6***	*ORF24*	exonuclease	16,251	17,162	4.98	34799.67	DNA Replication, repair, and recombination	*Yersinia* phage phiYeO3-12	NP_052100	99.3	0
***6.3***	*ORF25*	hypothetical protein	17,144	17,257	9.69	4111.08	unknown	*Yersinia* phage phiYeO3-12	NP_052102	97.3	8.79^e-15^
***6.5***	*ORF26*	hypothetical protein	17,401	17,595	6.57	7458.47	unknown	*Yersinia* phage phiYeO3-12	NP_052103	100	6.28^e-38^
***6.7***	*ORF27*	hypothetical protein	17,600	17,851	9.13	8833.96	unknown	Enterobacteria phage T3	NP_523330	98.8	1.76^e-49^
***7.3***	*ORF28*	tail assembly protein	17,879	18,199	9.78	11003.70	scaffolding protein required for the assembly of tail fibers on capsids	*Salmonella* phage phiSG-JL2	YP_001949779	95.3	2.72^e-39^
***8***	*ORF29*	head to tail joining protein	18,210	19,817	4.54	58649.37	bacteriophage head to tail connecting protein	*Yersinia* phage phiYeO3-12	NP_052106	100	0
***9***	*ORF30*	capsid assembly protein	19,919	20,851	4.24	33787.57	scaffolding protein required for the formation of pro-capsids.	*Salmonella* phage phiSG-JL2	YP_001949781	98.4	0
***10A***	*ORF31*	major capsid protein	21,008	22,054	7.11	36954.20	scaffolding protein	*Yersinia* phage phiYeO3-12	NP_052108	99.1	0
***10B***	*ORF32*	minor capsid protein	22,066	22,188	6.10	4295.84	scaffolding protein	*Salmonella* phage phiSG-JL2	YP_001949782	95.0	2.23^e-15^
***11***	*ORF33*	tail tubular protein A	22,270	22,860	4.48	22233.69	required for assembly of tails of T7-like phages	*Yersinia* phage phiYeO3-12	NP_052110	100	1.00^e-141^
***12***	*ORF34*	tail tubular protein B	22,876	25,281	6.11	89771.51	required for assembly of tails of T7-like phages	*Yersinia* phage phiYeO3-12	NP_052111	98.1	0
***13***	*ORF35*	internal virion protein A	25,354	25,779	5.37	16473.96	forms internal core of virion	*Salmonella* phage phiSG-JL2	YP_001949786	96.3	5.14^e-93^
***13.5***	*ORF36*	hypothetical protein	25,766	26,155	9.02	14582.99	unknown	*Yersinia* phage phiYeO3-12	NP_072072	73.6	2.87^e-59^
***14***	*ORF 37*	internal virion protein B	26,158	26,751	8.66	21308.02	forms internal core of virion	*Yersinia* phage phiYeO3-12	NP_052114	96.4	1.54^e-133^
***15***	*ORF38*	internal virion protein C	26,754	28,997	5.47	85134.48	forms internal core of virion	*Salmonella* phage phiSG-JL2	YP_001949788	99.3	0
***16***	*ORF39*	internal virion protein D	29,016	32,978	8.41	143525.96	forms internal core of virion	*Yersinia* phage phiYeO3-12	NP_052116	98.9	0
***17***	*ORF40*	tail fiber protein	33,050	34,996	6.45	69728.57	host recognition binding protein	*Yersinia* phage phiYeO3-12	NP_052117	89.3	0
***17.5***	*ORF41*	holin	35,008	35,211	6.08	7360.50	holin, class II	*Yersinia* phage phiYeO3-12	NP_052118	94.0	2.79^e-37^
***18***	*ORF42*	DNA packaging protein A	35,215	35,481	4.70	9888.31	DNA packaging	*Salmonella* phage phiSG-JL2	YP_001949792	100	1.01^e-55^
***18.5***	*ORF43*	Phage λ Rz-like lysis protein (Rz/Rz1 equivalent)	35,570	36,022	9.41	16993.28	host lysis (via Rz/Rz1 spanins disrupting outer membrane)	*Salmonella* phage phiSG-JL2	YP_00194793	98.7	4.69^e-105^
***19***	*ORF44*	DNA packaging protein B	35,997	37,760	5.32	66704.05	DNA packaging	*Yersinia* phage phiYeO3-12	NP_052122	99.8	0
***19.5***	*ORF45*	hypothetical protein	38,005	38,154	7.87	5441.55	unknown	*Yersinia* phage phiYeO3-12	NP_052125	98.0	1.78^e-24^
**-**	*-*	regulatory element	38,412	38,646	-	-	terminal repeat	-	-	-	-

Genes are listed by number, along with their predicted function, if known, followed by the nature of the evidence that supports the functional classification. Genes with no functional prediction, but with significant (E <10^-5^) sequence similarity to genes in the NCBI database as determined by BLASTP are listed, including the name of the organism in which the similar gene was found.

### Nucleotide metabolism, DNA replication and recombination

In the AP5 genome, at least eleven genes were identified that play a role in nucleotide metabolism, DNA replication, and recombination. The transcribed genes function to overcome host restriction and to convert the metabolism of the host cell to the production of phage proteins. The product of gene *0.3* is a small protein, which mimics B-form DNA, and binds to and inhibits type I restriction endonucleases [28–30], as well as possessing S-adenosyl-L-methionine hydrolase (SAMase) activity acting to degrade the methyl group donor and the methylation activities present in the host [31]. Dam (DNA adenine methyltransferase) methylase modifies GATC, and Dcm (DNA cytosine methyltransferase) methylase modifies CC(A/T)GG sequences [9]. As in *Yersinia* phage φYeO3-12, the sequences corresponding to restriction enzyme recognition sites GATC and CC(A/T)GG, are underrepresented in phage AP5 DNA, occurring only 4 and 3 times, respectively. Gene *0.7* codes for a protein kinase involved in host transcription shutoff [32] and phosphorylates host elongation factors G and P and ribosomal protein S6 [33]. Other genes include an RNA polymerase (gene *1*), a deoxyguanosine triphospho-hydrolase inhibitor (gene *1.2*), a DNA ligase (gene *1.3*), a host RNA polymerase inhibitor (gene *2*), a ssDNA binding protein/helix destabilizing protein (gene *2.5*), an endonuclease (gene *3*), a primase/helicase (gene *4*), a DNA polymerase (gene *5*), a host recBCD nuclease inhibitor (gene *5.9*), and an exonuclease (gene *6*).

### DNA packaging and morphogenesis

Several genes were identified that play a role in morphogenesis and DNA packaging. We identified two CDSs which display sequence similarity to the capsid proteins of phages belonging to T7-like viruses. The upstream gene *10A* displays homology to *Yersinia* phage φYeO3-12 major capsid protein 10A [NP_052108], while the downstream gene *10B* is similar to the minor capsid protein 10B in *Salmonella* phage φSG-JL2 [YP_001949782]. Some T7-like phages display two “versions” of the major capsid protein, which are designated as 10A and 10B [34]. The sequences of the amino termini of these proteins are identical, but during translation a -1 ribosomal frameshift allows for alternative reading frames within one mRNA, permitting the elongation of the protein product. The features of this system are a slippery site in the DNA/RNA and a downstream stem-loop structure capable of forming a pseudoknot [35, 36]. Analysis of AP5 using pKiss [37] did not yield evidence for a potential pseudoknot. Gene *9* was identified as the capsid assembly protein required for the formation of procapsids. The structure of this phage is therefore made up of gene *10A* and gene *10B* (capsid), the head to tail joining protein (gene *9*), and an internal core formed by the products of gene *13* (internal virion protein A), gene *14* (internal virion protein B), gene *15* (internal virion protein C), and gene *16* (internal virion protein D). These proteins are homologous to those that form the internal core of the T7 virion. In T7, along with internal virion proteins B and C, the internal virion protein D, is ejected from the phage head and forms part of a putative channel that spans the entire host cell envelope and allows entry of DNA. The N-terminus of this protein has similarity to a lytic transglycosylase and may help form a channel for phage DNA translocation through the peptidoglycan layer of the host envelope [18]. BLASTN analysis of gene *16* (internal virion protein D) confirms the presence of a peptidoglycan hydrolase motif at the N-terminus. Gene *7.3* was identified as the tail assembly protein required for assembly of tail fibers on capsids. Genes *11* and *12* correspond to tail tubular proteins A and B respectively required for assembly of tails. Gene *17*, codes for the tail fiber protein or host recognition binding protein and shares 89.3% identity with gp17, the tail fiber protein of *Yersinia* phage φYeO3-12 [NP_052117], and only 67% identity with gp17 of *Salmonella* phage φSG-JL2 [YP_001949790]. As with other gp17 homologs, sequence similarity is only found at the N-terminus, the part of the protein that is associated with the tail structure. The C-terminus is involved in ligand interactions and exhibits considerable differences, despite that phage AP5 shares a similar host range with *Yersinia* phage φYeO3-12 [9]. The large and small terminase subunit homologs were determined to be the products of gene *18* (DNA Packaging Protein A) and gene *19* (DNA Packaging Protein B).

### Host cell lysis

The final stage of the phage lytic cycle is degradation of the bacterial cell wall and release of progeny phages. The lysis of the cell wall is typically induced by two phage encoded proteins, a holin and an endolysin [38]. Endolysins are muralytic enzymes produced by dsDNA phages, which hydrolyze the peptidoglycan layer of bacterial cell walls. As in other T7 phages, gene *3.5* of phage AP5 is proposed to be the endolysin protein since it possesses N-acetylmuramoyl-L-alanine amidase activity. Access of endolysins to the cell wall occurs through the presence of a secondary lysis factor, known as a holin. Holins are usually small proteins characterized by the presence of transmembrane domains (TMD) [39]. The predicted proteins of AP5 were scanned for TMDs using TMHMM [40]. TMDs were identified in gene *0.6*, gene *6.3*, gene *17.5*, and gene *19.5*, which code for small proteins of 67, 37, 67, and 49 amino acids, respectively. The derived protein from gene *17.5* of AP5 is proposed as a holin since it is a small protein containing an N-terminal TMD and shares sequence similarity to *Yersinia* phage φYeO3-12 lysis protein [NP_052118]. Phage AP5 has also one more lysis gene (gene *18.5*) coding for a phage λ Rz-like lysis protein (PHA00276), an i-spanin of 150 amino acids which presents 98.7% sequence identity to λ Rz-like protein [YP_00194793] in *Salmonella* phage φSG-JL2. Further inspection of the gene *18.5* sequence, confirms the presence of a nested ORF of 255 bp (in the +1 reading frame) embedded entirely within the sequence coding for an o-spanin with homology to *Rz1* (*18.7*) of bacteriophage T7. Based on these observations, gene *18.5* is proposed as an *Rz/Rz1* equivalent lysis gene coding for transmembrane spanins involved in the disruption of the outer membrane of the host [41].

### Transcriptional and regulatory sequences

Phage AP5 was not found to contain tRNA genes, which is not an unexpected observation since no T7-like phages have been found to harbour them. A promoter was identified at position 550–580 bp of the genome with sequence similarity to host promoter consensus TTGACA(N15-18)TATAAT with a 2 bp miss-match suggesting the early genes of this type of virus are transcribed by the host RNA polymerase. This is a major dissimilarity between phage AP5 and T3/T7 phages where the latter possess multiple strong promoters recognized by the host RNA polymerase. As with all T7 group phages, the AP5 phage encoded RNA polymerase (RNAP), is responsible for the recognition of phage specific promoters. In phage AP5, we identified 14 phage-specific promoters using PHIRE [42], which are named according to the downstream gene (Table 3). The promoter sequences lie within intergenic regions and show the greatest similarity to those of *Yersinia* phage φYeO3-12 and bacteriophage T3.

**Table 3:** **Predicted promoter sequences of AP5**

Name	Promoter sequence	Number of mismatches ^†^	Transcription	
			Beginning	End
ϕGene *0.3*	AATTACCCTCACTAAAGGGAAT	4	475	496
ϕGene *1.05*	ATTAACCCTCACTAACGGGAGA	1	5970	5991
ϕGene *1.1*	GTTAACCCTCACTAACGGGAGA	2	6312	6333
ϕGene *1.3*	AATAACCCTAACTAACAGGAGA	4	6823	6844
ϕGene *1.6*	ATTAACCCTCACTAACAGGAGA	2	8015	8036
ϕGene *2.5*	AATTACCCTCACTAAAGGGAAC	4	9227	9248
ϕGene *4*	ATTAACACTCACTAAAGGGATG	3	11000	11021
ϕGene *4.3*	ATTAACCCTCACTAACGGGAAC	3	12818	12839
ϕGene *6.5*	ATTAACCCTCACTAAAGGGAAG	2	17277	17298
ϕGene *9*	AATAACCATCACTAAATGGAGA	3	19816	19837
ϕGene *10A*	ATTAACCCTCACTAAAGGGAGA	0	20851	20872
ϕGene *13*	ATTAACCCTCACTAAAGGGAGA	0	25303	25324
ϕGene *17*	ATAAACCCTCACTAAAGGGAGA	0	32975	32996
ϕGene *19.5*	ATTAACCCTCACTAAAGGGAGA	0	37842	37863
	*Consensus sequence*			
AP5	ATTAACCCTCACTAAAGGGAGA			
φYeO3-12	ATTAACCCTCACTAAAGGGAGA			
T3	ATTAACCCTCACTAAAGGGAGA			
T7	TAATACGACTCACTATAGGGAG			

^†^Number of mismatches compared to AP5 consensus sequence.

## Conclusions

In this manuscript we have reported on the morphology and genome of the phage vB_YenP_AP5. Due to its lytic nature and marked specificity to *Y.enterocolitica* strains of serotype O:3, this phage is a potential biotechnological tool for diagnostic, therapeutic, and/or bio-control uses, given that O:3 is the most predominant serotype involved in human food-borne infections [4]. Additionally, the genome of this phage does not contain any undesirable laterally transferable genes that are related to bacterial toxins, pathogenicity, antibiotic resistance and/or lysogeny on the basis of homologies with known virulence and resistance genes available in GenBank.

## Methods

### Bacterial strains and growth media

Tryptic Soy Broth (TSB), Tryptic Soy Agar (TSA), and Tryptic Soft Agar (TSB +0.6% agar) (Difco Laboratories, Detroit, MI) were used to grow the host bacteria and to propagate the phage_._ In procedures involving phage infection, media were supplemented with filter-sterilized CaCl_2_.2H_2_O to a final concentration of 5 mM. *Y. enterocolitica* strains of serotype O:3 were used as indicator strains for phage isolation (Table 1)*. Y. enterocolitica* 6471/76-c of bioserotype 4/O:3 [43] obtained from the Félix d’Hérelle Reference Center for bacterial viruses (Université Laval, QC, Canada) was used for phage propagation. *Y. enterocolitica* 6471/76, *Y. enterocolitica* strains of serotype O:1 and O:2, and *Y. enterocolitica* O:3 mutants, were acquired from the Haartman Institute, University of Helsinki, Finland. Other strains from the genus *Yersinia* were obtained from the Ontario Agency for Health Protection and Promotion (OAHPP) (Ontario, Canada), and the American Type Culture Collection (ATCC) (Manassas, Virginia, USA).

### Isolation and propagation of phage

A 1 L sample of preliminary treated sewage from a local water treatment plant (Guelph, Ontario) was centrifuged twice at 10,000 *g* for 20 minutes at 4°C using a Beckman high-speed centrifuge and a JA-10 fixed-angle rotor (Beckman, Palo Alto, CA, USA) and the supernatant passed through a sterile filter membrane of 0.45 μm pore size (Fisher Scientific, Mississauga, ON, Canada). Equal 9 ml volumes of the filtered supernatant and TSB were inoculated with 200 μL of an overnight mixed culture of selected *Y. enterocolitica* strains of serotype O:3 and incubated for 18–24 h at 30°C with gentle shaking. After incubation, the enrichments were centrifuged at 10,000 *g* for 20 minutes at 4°C and the supernatant filtered through a sterile disposable filter of 0.45 μm pore size, and the filtrates stored at 4°C. Phages were detected by spot tests [16] on indicator strains incubating for 16–20 h at 25°C. Complete or partial lysis zones were then removed by cutting the soft layer from the plates using a sterile pipette tip and placing them separately in 1 mL of SM buffer (5.8 g of NaCl per liter, 2.0 g of MgSO47H2O per liter, 50 mM Tris–HCl [pH 7.5]), and used in standard double agar overlay plaque assays [43] to identify plaques showing different size and plaque morphology. Three rounds of repeated single plaque isolation were then performed to ensure unique phages were obtained. Purified phages were named following the naming convention of Kropinski et al. [44]. The small drop plaque assay was used to determine the titer of phage preparations [45].

### Host range determination

The lytic activity of vB-YenP-AP5 was tested against 60 *Yersinia* strains as determined by standard spot tests [16]. Briefly, 10 μl from a purified phage suspension containing approximately 10^8^ pfu/mL were spotted in the middle of a lawn of bacteria and left to dry before incubation for 18–24 h. Each strain was tested three times at 25°C and at 37°C. The degree of lysis was recorded using a four-point scale: (+4) complete clearing, (+3) clearing throughout but with a faint hazy background, (+2) substantial turbidity throughout the cleared zone, and (+1) a few individual plaques.

### Transmission electron microscopy

The phage was pelleted at 25,000 × g for 1 hour at 4°C, using a Beckman high-speed centrifuge and a JA-18.1 fixed-angle rotor (Beckman, Palo Alto, CA, USA). The phage pellet was washed twice under the same conditions in neutral 0.1 M ammonium acetate [46] *.* The final phage sediment was re-suspended in 150 μL of SM-buffer supplemented with 5 mM CaCl_2_. Samples were then deposited onto carbon-coated Formvar films on copper grids, and stained with 2% uranyl acetate (pH 4) or 2% potassium phosphotungstate (PT, pH 7.2), air dried, and examined under a Tecnai G2 F20 transmission electron microscope (FEI, Hillsboro, OR, USA), operating at 120 KEv. Images were collected and analyzed using Digital Micrograph™ Software (Gatan, Pleasanton, CA, USA).

### Isolation of phage DNA

To separate phage from bacterial debris, a crude phage lysate was centrifuged at 10,000 × g for 15 min at 4°C and the supernatant filtered through 0.22 μm low protein binding filter (Millipore, USA). Contaminating nucleic acids in the supernatant were digested with pancreatic DNase 1, and RNase A, each added to obtain a final concentration of 10 μg/mL (Sigma-Aldrich Canada Ltd., Oakville, ON), for 15 min at room temperature. DNA isolation was then performed with a commercial Phage DNA Isolation Kit (Norgen BioTek Corp.,Thorold, ON., Canada), as per the manufacturer’s instructions. The DNA was characterized spectrophotometrically.

### Genome sequencing and assembly

Phage genomic DNA was fragmented using Ion Xpress™ Plus gDNA Fragment Library kit following the manufacturer’s protocol (Life Technologies, Foster City, CA). The fragmented DNA was collected using Pippin Prep DNA Size Selection System (Sage Science, Beverly, MA) and assessed for concentration and size distribution using a Bioanalyzer 2100 (Agilent Technologies, Mississauga, ON). The DNA fragments were then attached to the surface of Ion Sphere particles (ISPs) using an Ion Xpress Template kit (Life Technologies) according to the manufacturer’s instructions. Template-ISPs were sequenced using 316 micro-chips using an Ion Torrent Personal Genome Machine (PGM) with an Ion PGM Sequencing 400 kit (Life Technologies). The sequence reads were filtered using PGM software to remove low quality sequences, trimmed to remove adaptor sequences and the filtered sequences were assembled. The assembled genome had a coverage of 33.4×. Gaps were identified using the Lasergene® Genomics Suite of DNAStar software (DNAStar Inc., Madison, WI). The gaps were closed by PCR using primers flanking regions adjacent to the gaps and sequencing using a 3730 Genetic Analyzer (Life Technologies). The final assembled genome was manually curated for errors.

### Bioinformatics analysis

The phage genome was analyzed for coding sequences using Kodon version 2.0 (Applied Maths Inc., Austin, TX, USA). Genes were identified from among the predicted coding sequences based on the presence of ATG, GTG, CTG or TTG start codons, followed by at least 30 additional codons, and an upstream sequence resembling the following ribosome-binding site, GGAGGT [47, 48]. A search for phage-encoded tRNA genes was performed with tRNAScan-SE and Aragorn, using default parameters [49, 50]. Preliminary annotation of genes was performed using myRAST [51]. Additional manual functional annotation was performed using the Geneious software version 7.1.5 (Biomatters) [52, 53]. Phage-specific promoters were discovered using PHIRE [42] using a length (L) of 22 bp and a degeneracy (D) of 4 bp. Determination of theoretical molecular weight and isoelectric point employed ExPASy via http://web.expasy.org/compute_pi/ [54–56]. BLASTP and Psi-BLAST algorithms were used to determine the similarity to described proteins in the National Center for Biotechnology Information [NCBI] database (http://www.ncbi.nlm.nih.gov). Whole genome comparisons were carried out using Mauve [26], and CoreGenes [24].

### Genome sequence

The annotated genome sequence for the phage vB_YenP_AP5 was deposited in the NCBI nucleotide database under the accession number KM253764.
